# Is the Memory Effect of the Blind Spot Involved in Negative Dysphotopsia after Cataract Surgery?

**DOI:** 10.1155/2015/786579

**Published:** 2015-09-06

**Authors:** Martin Wenzel, Rupert Menapace, Timo Eppig, Achim Langenbucher

**Affiliations:** ^1^Eye Clinic Petrisberg, Max-Planck-Straße 14–16, 54296 Trier, Germany; ^2^University Eye Clinic, Währinger Gürtel 18-20, 1090 Vienna, Austria; ^3^Department of Experimental Ophthalmology, Saarland University, Kirrberger Straße 100, Building 22, 66424 Homburg/Saar, Germany

## Abstract

We present novel clinical observations on negative dysphotopsia (ND) in eyes that have undergone cataract surgery. In the past, shadow effects were alleged to be located in the far peripheral temporal visual field 50° to 100° away from the optical axis. In a small series of eight patients we found evidence of photic effects, described by the patients as shadows in the periphery that were objectively located much more centrally. In all cases, we could find an association of these phenomena with the blind spot. We hypothesize that the memory effect of the blind spot which is dislocated and changed in magnification due to replacement of the crystalline lens could be one determinant for pseudophakic ND. The scotoma of the optic nerve head and the main arteries and veins of the phakic eye are displaced in the pseudophakic eye depending on the specific characteristics and position of the intraocular lens within the eye.

## 1. Introduction

In 2000, Davison was the first to focus on a relatively common problem after uncomplicated cataract surgery, which was called negative dysphotopsia (ND) [1]. This side effect of cataract surgery typically vanishes after a couple of weeks or months and rarely persists for longer periods of time. In most cases it does not have serious clinical consequences. Since Davison, these photic effects have been extensively discussed in the literature, but up to now there is no generally accepted concept which could describe causality of ND and describe the symptoms of the patients [2–8]. The ND phenomena refer to arc or bow shaped high contrast shadows in the temporal visual field, which appear in the early time after cataract surgery and disappear in most cases after a couple of weeks. In some cases, however, they persist for a longer time period and patients are severely disturbed and insist on surgical interventions such as intraocular lens (IOL) explantation or implantation of an add-on IOL. Up to now, only F. F. Marques and D. M. V. Marques quantified these optical phenomena clinically by performing visual field measurements [9]. They described a scotoma in the superior-temporal visual field at an eccentricity of about 20°, which improved upon pharmacological miosis. Some authors who addressed negative dysphotopsia reported these shadows to appear mainly in the far periphery 50° to 100° away from the fovea [2, 6, 10]. Davison [1], Holladay et al. [2], Osher [5], and Trattler et al. [10] published patient drawings or sketches of these light and shadow effects without proof of their exact localization in the visual field. Holladay et al. and Hong et al. investigated the causes of ND by means of optical simulations [2, 7]. The published literature [1, 5] reports an incidence between 0.2% and 20%. Photic effects were first described with PMMA lenses [11–13]; however, most authors reported ND with acrylic or silicone intraocular lenses [1, 5, 9, 10, 14–20].

## 2. Materials and Methods

A total of 800 cataract procedures were performed between December 2014 and February 2015. All patients underwent cataract surgery under local topical anesthesia with implantation of foldable acrylic single-piece monofocal hydrophobic intraocular lenses (Acrysof SA60AT or SN60WF, optic diameter of 6 mm) through a 5.5 to 6 mm capsulorhexis. The overlap of the anterior capsular remnant over the anterior optic surface was less than 0.5 mm in all cases. In all patients cataract surgery was uneventful and all IOLs were well centered in the capsular bag.

Directly after cataract surgery some patients reported on sharply bounded shadows in the temporal visual field which were majorly disturbing in every day's life.

For localization of the shadow in the visual field, we used the physiological scotoma corresponding to the blind spot. If the location of the blind spot (approximately 15° temporal to the visual axis) in the visual field is known, the location of the photic phenomena could be referenced in the visual field.

**Figure 1:**
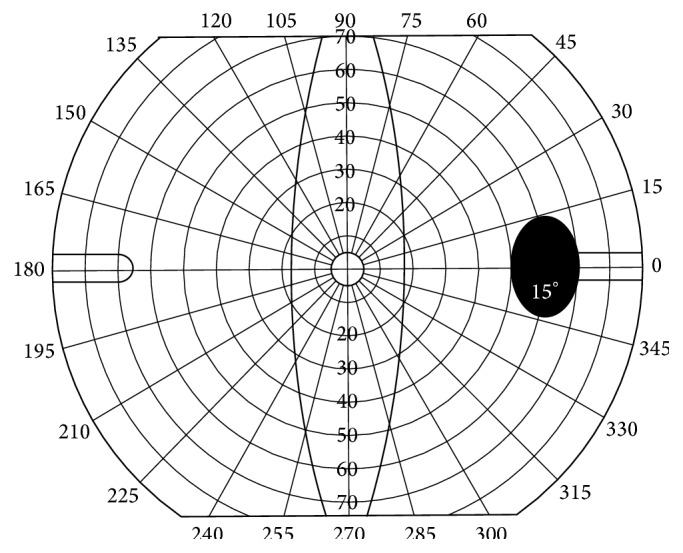
Dimension of the blind spot in a scale of Figure 2.

Patients were positioned at a desk in reading position and a standard preprinted form sheet, normally used for Goldmann perimetry, was presented to the treated eye while the contralateral eye was occluded. At the intersection of the horizontal meridian with the 60° mark of the visual field a red spot was drawn, which referred to the blind spot. Due to the short distance of the paper form and the resulting magnification, the mark at 60° corresponded to 15° in the visual field under test conditions (Figure 1). The sheet was positioned on the desk so that the center was located straight in front of the eye and the red spot was located temporally. Patients were requested to monocularly fixate the center of the scheme while moving the head slowly back and forth until the red spot disappeared. The measurement distance when the red spot disappeared was typically 25 cm. Then, patients were asked to mark the shadow on the sheet while keeping the head position and fixation unchanged while the red spot had to remain invisible. If they had located the black shadow outside the scheme, we would have extended the form sheet laterally to enlarge the drawing canvas.

## 3. Results and Discussion

Photic effects were reported in one percent (8 out of 800) of our treated eyes. Patient data and biometric and functional results of these eyes are summarized in Table 1. The refractive error before and after surgery did not exceed ±1 D. No pathology could be found, which could explain the patients' complaints.

At the beginning of the follow-up examination the patients were asked to suspect where the black shadows were located within the visual field. All eight patients reported phenomena within the peripheral visual field between 30° and 100°.

We asked our patients to sketch the position of the shadow according to the above-mentioned method.

**Figure 2:**
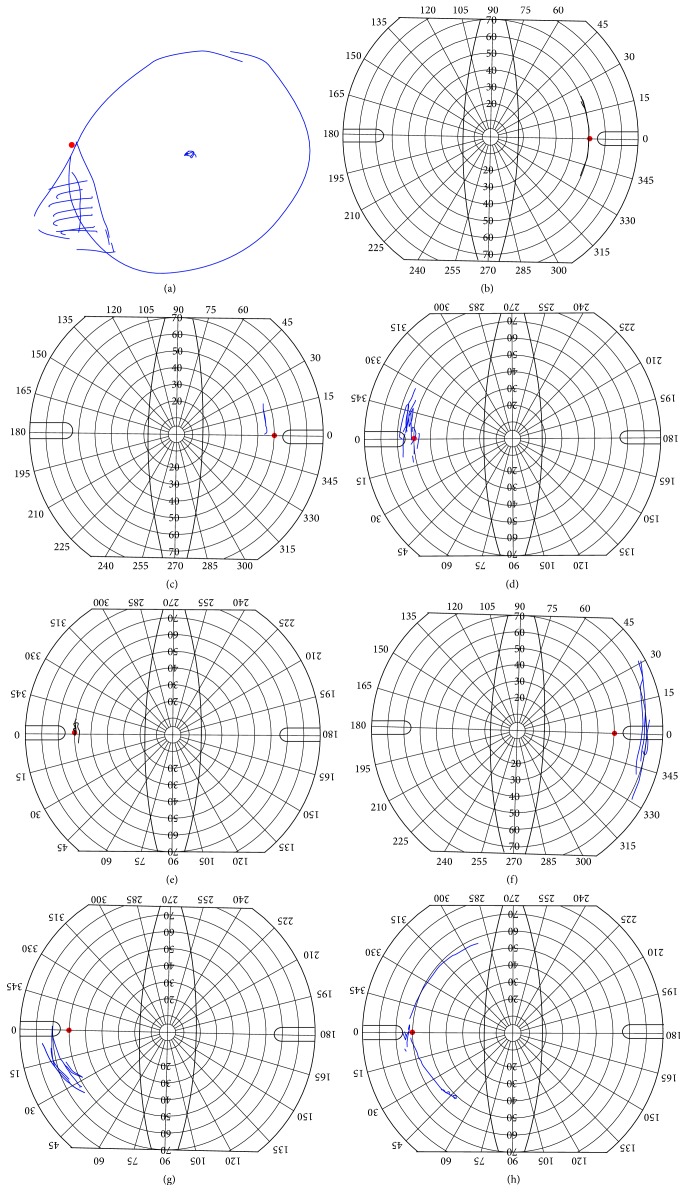
Patient drawings of shadows in the visual field (negative dysphotopsia) up to 5 months after cataract surgery. Due to the measurement distance, all dimensions have to be divided by 4. The blind spot is typically located 15° temporal to the visual axis (60° in the form sheet).

Most of the eight patients had difficulties in fixating the center and mark the shadow effects outside the center of the visual field scheme. Finally, all of them marked the shadow directly adjacent to the blind spot (Figure 2). None of the patients located their ND more than 20° away from the center. This result was surprising for both the patient and the examiner as the shadow was expected to be located much more in the periphery.

This subjective examination technique shows some limitations: we estimate the inherent accuracy to be about 5°. The small red dot used for localization of the blind spot is only about 1°, whereas the blind spot has an extent of about 5°. Thus, the exact location of the red spot within the blind spot is not possible. This explains why scotoma between 10° and 20° temporally located from the central fixation can still be related to the blind spot, as depicted in patient #6.

Up to now, the etiology of ND is only understood if the photic effects are located in the extreme periphery [6, 7]. We do not believe that a black shadow outside the center at 90° would disturb the patient seriously. Peripheral defects in the visual field caused by glaucoma are also rarely perceived as disturbing. The shadow with the bird published by Osher [5] does more seem to be at about 15° than 90° out of the center. We interpret our results in that way that ND is multifactorial and one reason could be a change of the location and size of the blind spot due to replacement of the crystalline lens by an artificial lens. If we assume that there might be a memory effect for the blind spot, changes of the position or size due to changes in the imaging channel could be responsible for these phenomena observed by the patients. This explanation maintains that in most cases ND vanishes after a couple of weeks or months spontaneously, whereas in other cases where the location or size changes are larger or the memory effect of the blind spot which is accompanied with the image processing in the brain works differently. The reaction to a change of location or size of the corresponding image to the blind spot is individually different, and in some cases this effect disappears rapidly whereas in others it persists over a long period of time. Any shift of the IOL optic, different optical design, decentration, and/or tilt changes magnification or position of the image on the retina. Consequently, additional implantation of a sulcus fixated lens does also change image magnification and location.

In general, most patients adapt themselves to the slightly different magnification after cataract surgery and do not suffer from visual discomfort. Only about 1% of the patients complain about temporary disorders such as ND. Such an adaptation process is often argued to explain the spontaneous occurrence and disappearance of ND. Others have described complete or partial disappearance of ND with IOL exchange, Nd:YAG laser treatment of anterior capsule overlap, reverse optic capture, and piggyback IOL implantation in some cases, whereas others report no effect of these subsequent procedures [21–27]. We assume that IOL exchange and piggybacking may lead to a change of magnification or repositioning of the IOL image effectuating the symptoms to disappear. In contrast, anterior capsulotomy or primary reverse optic capture may be useful to treat or prevent ND caused by an anterior capsule overlap as supposed by Hong et al. and Masket and Fram [7, 27].

We counsel the patients with our hypothesis that a minor change in object-image magnification of the pseudophakic eye with respect to the phakic eye might change the location or size of the corresponding image to the optic disc head which might result in an arc-shaped shadow adjacent to the blind spot. Our patients were generally satisfied with this explanation and did not insist on an additional surgical intervention. Follow-up examinations are necessary to verify our hypothesis on ND and whether these shadows will disappear on temporary occlusion of the eye.

## 4. Conclusions

Up to now, pseudophakic negative dysphotopsia was alleged to be located in the far peripheral temporal visual field between 50° and 90°. We assume that changes in the location and/or size of the blind spot in the visual field (as a corresponding image to the optic nerve head in the object plane) could be one reason for photic effects, which were described excessively in the literature as pseudophakic negative dysphotopsia. In that context, we present a new examination technique to localize the photic effects within the visual field in patients with negative dysphotopsia.

In all of our 8 patients, the photic effects were associated with the blind spot. The scotoma of the optic nerve head and the main arteries and veins of the phakic eye are displaced in the pseudophakic eye depending on the specific IOL position, power, and shape factor. We feel that clinicians should keep in mind that beside the edge design or reflectance of IOLs or the direct peripheral pass of light without being refracted by the IOL there might be other reasons, for example, associated with the blind spot which could cause photic effects in the pseudophakic eyes and which are typically located more centrally in the visual field.

## Figures and Tables

**Table 1:** Clinical data of 8 patients with negative dysphotopsia.

Patient	Age	VA	Eye	IOP	AL	ACD	CR	IOL	VApost	Month
a	66	0.6	L	24	23.1	2.5	7.6	22.5	1.0	4
b	58	0.6	R	16	24.9	3.6	8.1	19.5	1.0	3
c	70	0.5	R	18	22.5	3.2	7.5	23.5	1.0	2
d	73	0.6	L	16	23.0	2.8	7.5	21.5	0.8	1
e	60	0.6	L	17	24.2	3.7	7.9	21.5	1.0	1
f	66	0.3	R	18	23.3	3.3	7.8	22.5	0.6	1
g	65	0.5	L	17	22.8	2.9	7.5	23.0	1.0	2
h	63	0.5	L	17	22.3	2.9	7.3	23.0	1.0	1

Age: age of the patient at time of surgery; VA: visual acuity (decimal) before surgery; eye: site of surgery; IOP: intraocular pressure before surgery; AL: axial length; ACD: anterior chamber depth before surgery; CR: corneal radius (mean); IOL: diopter of the implanted IOL; VApost: visual acuity (decimal) after surgery; month: time of examination after surgery.
